# Initiating Care of a Patient With Myalgic Encephalomyelitis/Chronic Fatigue Syndrome (ME/CFS)

**DOI:** 10.3389/fped.2018.00415

**Published:** 2019-01-23

**Authors:** Charles W. Lapp

**Affiliations:** Hunter-Hopkins Center PLLC, Charlotte, NC, United States

**Keywords:** myalgia, encephalomyelitis, chronic fatigue, patient care, management—healthcare

## Abstract

This paper introduces the primary care physician to the unique and challenging aspects of initially diagnosing and managing a complex condition for which there are a plethora of symptoms, few physical findings, no known cause, and no specific treatments. While daunting, the rewards are many, and those who pursue an interest in ME/CFS find themselves at the forefront of medicine.

The approach to any complex problem is to break it down into small steps, and ME/CFS is no exception. The first office visit should be devoted to a history of the present illness, a physical examination, and collection of exclusionary laboratory tests. On follow-up the differential diagnosis and a treatment plan can be addressed. Many individuals with ME/CFS have been humiliated or dismissed by other providers, so one will need to be as non-judgmental as possible and acknowledge that ME/CFS is not a psychological condition but a real illness. They need reassurance that you will work with them to seek a unifying diagnosis and prioritize management.

## History of the Present Illness

At the start of the interview process it is helpful to know the patient's chief concerns, focusing at first on the major symptoms. Since most patients are seeking to confirm a diagnosis of ME/CFS, you may wish to clarify the core symptoms (1). By “**fatigue**” does the patient mean a lack of energy and stamina, or more sleepiness? If the former, does he or she describe exertion intolerance and post-exertional malaise after over-exertion? Is this fatigue severe enough to markedly affect lifestyle, work or educational activities? Is there **chronic widespread pain**? If so, is it severe enough to affect mood, mobility, and sleep? Is he or she experiencing new or different **headaches**? Does the patient report significant problems with **attention, concentration, comprehension, short term memory** loss, recall, multitasking, distractibility, forgetfulness, difficulty with mental math (like making change or calculating a tip), disorientation or confusion? Are there changes in the sleep pattern or **non-restorative sleep**? Is he or she noting **orthostatic dizziness**, “stars,” tunnel vision, or feeling **uncomfortable standing in place**?

In addition to these core symptoms of ME/CFS, many patients have co-morbidities including fibromyalgia, irritable bowel syndrome, overactive bladder, sicca syndrome (dry eyes and mouth), dysautonomia, and others (Table 1). Thus, a detailed review of symptoms is important, and a large list of co-morbidities is supportive of the diagnosis.

**Table 1:** ME/CFS overlap syndromes.

These co-morbidities have been associated with ME/CFS or occur more commonly in Persons with Chronic Fatigue Syndrome (PWCs) than the general population: Fibromyalgia Myofascial Pain Syndrome Irritable Bowel Syndrome Overactive bladder or Interstitial Cystitis Postural Orthostatic Tachycardia Syndrome Neurally Mediated Hypotension Mast Cell Activation Syndrome Hypogonadism and premature menopause Sleep disorders (sleep apnea, myoclonus / PLMS, non-restorative sleep) Restless Leg Syndrome / Periodic Leg Movement syndrome Multiple Chemical Sensitivities Hypersensitivities to light, sound, smell, touch, chemicals or odors Sacroiliac joint tenderness Hypoglycemia Mitral valve prolapse Premenstrual Syndrome or Premenstural Dysphoric Disorder Allergies Vasomotor (autonomic or non-allergic) rhinitis B12 deficiency Gut motility disorder with dysphagia, early satiety, nausea, and/or constipation Celiac- or sprue-like disorders with sensitivity to wheat, grains, or gluten Autonomic dysfunction with low blood pressure, orthostatic symptoms Sicca complex or Sjogren's syndrome Bronchostriction (reactive airways or asthma) Macrocytosis (large red cells, causes low sedimentation rate) Abdomino-pelvic pain Vulvodynia or vulvar vestibulitis Joint hyperlaxity with or without hyperelasticity (Ehlers-Danlos Stigmata) Milk protein intolerance Costochondritis Endometriosis Metabolic Syndrome Temperomandibular Dysfunction

In 2015, the Institute of Medicine provided a simple and practical diagnostic tool that practitioners might find helpful for screening (1):

The diagnosis requires that the patient have the following three symptoms:

A substantial reduction or impairment in the ability to engage in pre-illness levels of occupational, educational, social or personal activities, that persists for more than 6 months and is accompanied by fatigue, which is often profound, is of new or definite onset (not lifelong), is not the result of excessive ongoing exertion, and is not substantially alleviated by rest.

Post-exertional malaise

Un-refreshing sleep

At least one of the two following manifestations is also required:

Cognitive impairment

Orthostatic intolerance

*The diagnosis of ME/CFS should be questioned if patients do not have these symptoms at least half of the time with moderate, substantial, or severe intensity*.

This is meant to be a preliminary diagnostic tool. If the diagnosis seems probable it is best to confirm it with a more detailed instrument such as the Canadian Consensus Criteria (Appendix A in Supplementary Material) or the 1994 International Diagnostic Criteria (Appendix B in Supplementary Material).

## Physical Examination

It has been said that the physical examination is mostly normal in ME/CFS, but a careful exam can reveal many clues to this illness. The physical is also used to exclude other plausible causes for fatigue and other symptoms:

- The resting heart rate is typically higher than normal and body temperature may be subnormal.
- Blood pressures tend to be low. It is important to check orthostatic heart rate and blood pressure supine, after 1–2 min standing, and again after 5 min standing since many individuals have orthostatic hypotension or tachycardia (POTS) (2). A smaller number have Neurally Mediated Hypotension, but this is a delayed phenomenon that usually does not occur before 15–20 min of upright posture (3).
- Slowly reacting, non-reacting, or unequal pupils may reflect parasympathetic imbalance.
- Check for temperomandibular joint tenderness or crepitus.
- Non-exudative pharyngitis with “crimson crescents” in the posterior pharynx is frequently seen (4).
- Check for cervical, occipital, and axillary lymphadenopathy or tenderness.
- Check for carotidynia.
- Muscle tension in the neck and shoulders will frequently cause a loss of cervical lordosis and/or forward (“sniffing”) posture.
- Right upper quadrant tenderness without guard or rebound is common.
- Tenderness of the sacroiliac joints is very common.
- Check for joint hyperextensibility (5).
- Perform a manual tender point examination for fibromyalgia tender points or have the patient complete the 2011 clinical fibromyalgia survey (6).
- Finger-to-nose and rapid alternating movements may reveal dysmetria, dysdiadokinesia, or tremor.
- Balance testing and tandem stance (10 s) is frequently tenuous in patients.
- Romberg testing is frequently abnormal and may correlate with illness severity (7).
- Check deep tendon reflexes. Asymmetry and clonus are significant.
- Is there acne rosacea, livido reticularis, or dependent rubor?
- Ask the subject to perform Serial 7 Subtractions and the Digit Span Test.

## Laboratory

Lab studies are mostly performed to exclude other plausible causes for fatigue and are generally unremarkable. The Centers for Disease Control and Prevention has recommended (8):

Complete blood count (CBC).
Comprehensive Metabolic Panel (electrolytes, BUN, creatinine, glucose, calcium, phosphorus, total protein, albumin, globulin, alkaline phosphatase, SGOT/ALT, SGPT/AST).
C-reactive protein or Westergren sedimentation rate.
Thyroid function tests.
TSH is *least* important due to HPA Axis suppression in ME/CFS.
Free T4 and/or total T3.
Urinalysis.

Additionally, obtain any other laboratory studies indicated by your history and exam, such as:
ANA, Rheumatoid Factor or anti-CCP antibodies.
Cranial MRI if Multiple Sclerosis or other neurological disorder suspected, although small T2 weighted high intensity white matter lesions are seen in about 80% of cases.
Overnight sleep study (primary sleep disorders such as apnea and periodic leg movement syndrome occur in up to 60% of patients).
Sjogren's antibodies [SSA (Ro)/SSB (La)] if dry eyes and mouth are present.
Lyme serology (ELISA) or Western Blot if patient has had tick exposure or comes from an endemic area (Northeast US, Wisconsin area, California, and others).
Hepatitis C serology if “at risk” or has had elevated liver function tests.
CPK if muscle tenderness is present and myositis is suspected.
Obtain consultation if a significant psychiatric condition is present or suspected.

## Exclusionary Conditions, Mimics, and False Diagnoses

A grave concern with ME/CFS is that the symptoms are so diverse that other conditions may seem to overlap, and one does not want to diagnose ME/CFS when another, perhaps treatable, condition is actually at fault. Of course, a diagnosis of ME/CFS should not be entertained if there is an active medical condition such as narcolepsy or thyroiditis that could plausibly explain the fatigue and other symptoms. Some disorders confound the diagnosis so profoundly that they exclude the diagnosis of ME/CFS. These include melancholic depression, bipolar depression, schizophrenia, frank psychoses, active eating disorders, alcohol and substance abuse. Individuals with a BMI > 40–45 actually experience so much fatigue that it is difficult to discriminate from ME/CFS. Conditions such as sleep apnea, sarcoidosis, inflammatory bowel disease, and rheumatic conditions can all cause fatigue but if they are treated and stable the fatigue and systemic symptoms are generally much less than experienced in persons with ME/CFS and would not be considered exclusions. If there is a questionable diagnosis, then a diagnosis of Idiopathic Chronic Fatigue should be made, and the patient followed periodically over time (Diagram 1 in Supplementary Material).

## Management

Many patients will be seeking rapid relief and even a cure for their illness, but foremost they must have realistic expectations: ME/CFS is a chronic illness for which there is currently no known cure. Nevertheless, there are many treatments that can be helpful to reduce symptoms and improve functionality.

Most experts would agree (9) that it is most important to address exertion intolerance and post-exertional malaise first; then sleep and pain, followed by cognition and the co-morbidities. Experts will agree that patients must avoid over-exerting and then flaring or relapsing—referred to as “pushing and crashing”—which clearly exacerbates the illness and hinders improvement. The controversy surrounds how to best prevent that.

One technique is interval activity or time-based activity. If an individual knows that they can be active for a period of time without triggering symptoms—say 15 min—then he or she can shop or work for 15 min, take a break, then shop or work for another 15 min, and so on. Over time, the activity interval can be increased (10).

Another technique is to monitor steps per day by wearing a step meter or pedometer (11). It is important for patients to take at least 1,000 steps per day in order to avoid deconditioning; but patients are encouraged to calculate their average steps per day during a good week with no flares or relapses. This is typically about 2,500–3,500 steps per day. They are then encouraged to not exceed that number of steps. So if a patient went shopping or sightseeing one day and reached her average limit of 3,500 steps, she would know to quit and rest as soon as possible to avoid a flare or relapse.

Scientific evidence is mounting that patients should not exceed their Anaerobic Threshold, an activity level at which the heart and lungs cannot supply enough oxygen to the mitochondria. In the absence of oxygen, glucose metabolism is much less efficient and produces lactic acid and other toxins that seem detrimental to our patients. The Anaerobic Threshold is usually determined by specialized exercise testing, but is related to one's heart rate. So if a patient can monitor heart rate, he or she can estimate the maximum heart rate (frequently under 110 in adults) that can be tolerated without triggering a flare. Then avoid exceeding that heart rate except for short periods (12).

In short, it is very important to balance any activity with generous amounts of rest. So the patient should be encouraged to remain active, but not so active as to trigger flares and relapses.

Sleep is the next most important area to address. Start with typical sleep hygiene principles. Patients may consider over-the-counter sleep aids such as melatonin, theanine, valerian, tryptophan, antihistamines (diphenhydramine, doxylamine), or proprietary sleep aids. Low dose tricyclic or tetracyclic antidepressants, cyclobenzaprine, or low dose tizanidine are frequently prescribed to maintain sleep. If necessary, consider prescribing the usual benzodiazepine -based sleep medications to initiate sleep. Between 18 and 62% of persons with ME/CFS have primary sleep disorders, so highly consider referral to a sleep specialist if a sleep disorder is suspected (13).

Pain is another major symptom to address as it may affect sleep, mood, mobility and other domains. First identify the sources of pain: Fibromyalgia? Myofascial pain? Headache? Arthralgia? Inflammatory joint pain? Then assess the patient's need for pain intervention. Will non-pharmacologic therapy suffice such as hot packs, cold packs, liniments, baths or showers, massage, chiropractic, acupuncture, or TENS? If pharmacologic therapy is indicated, have non-opioid therapies been tried such as Cymbalta/duloxetine, Savella/milnacipran, or Lyrica/pregabalin? (7) In the last 10 years Low Dose Naltrexone has become a primary consideration in opioid-naïve individuals (14). If opioid medications are indicated, tramadol has been very effective, but many providers would be most comfortable referring to a pain specialist for anything more potent. In the case of migraine or rheumatic pain, specialists might also be indicated.

Cognitive problems tend to wax and wane, much as fatigue does. Patients need to be reassured that they are not developing Alzheimer's or dementia, and there is no evidence that such cognitive losses are permanent. While medication has helped little to improve cognition, the provider can suggest helpful techniques such as:

- Keep a calendar, notebook and calculator at hand.
- Always carry a cell phone to call for assistance, use as a GPS, or photograph your location in a parking lot or unfamiliar area.
- Develop the habit of always putting up important items such as keys, purses, wallets, and glasses in the same place.
- Plan important tasks to be done during the “best time of your day.”
- Avoid chaotic, stressful, or multisensory situations or events.

Autonomic, (auto)immune, (neuro)endocrine, psychological and co-morbid issues are managed as you would normally in your medical practice. It is imperative to address co-morbidities because they confound the ME/CFS. Consultants may be required. It is very important not to attribute all new symptoms to ME/CFS alone. Lastly, patients must maintain adequate hydration and nutrition although they tend to neglect these areas due to fatigue.

## Disability

It is estimated that more than 50% of persons with ME/CFS are disabled and up to 75% are unable to work or attend school regularly (1), so many patients may want to discuss the possibility of obtaining disability. Those who have a private disability contract are bound to the terms of that contract, and need to apply through their human resources department at work or directly to the insurer. If the policy is work-related it will probably be governed by ERISA regulations, which are very stringent. In that case, the individual is highly recommended to seek the advice of an ERISA-knowledgeable attorney. Those who apply for Social Security should check with their local Social Security office first to assure that they qualify with enough work credits. One must apply for Social Security within 5 years of stopping work. Patients should expect an initial rejection of their claim and then move on to an appeal and a hearing before an Administrative Law Judge. Attorney representation is strongly urged here.

Regardless of the type of disability insurance, documentation of disability will be key. The provider must document any inability to perform Activities of Daily Living (ADLs: bathing, dressing, feeding oneself, toileting, etc.) and Instrumental Activities of Daily Living (IADLS: cooking, housekeeping, shopping, laundering, socializing, managing finances, driving, traveling). In addition it is important to comment on physical abilities to lift, carry, sit, walk, or stand; and how often is the patient homebound or bedbound. This information can be obtained informally during the interview or by using checklists and forms at each visit.

Regardless of such testimony, objective evidence is most important, so supportive findings on the physical exam (especially orthostatic hypotension or tachycardia on the “stand test,” Fibromyalgia tender points, abnormal neurological findings), abnormalities on the cranial MRI, high titers of EBV VCA-IgG and/or EBV Early Antigen, are very helpful. If available, neuropsychiatric testing, tilt table testing, and 1- or 2-day Cardio-Pulmonary Exercise Testing can be extremely supportive.

## Making Time for the Patient

Persons with ME/CFS (PWCs) are admittedly very complex and challenging but eternally grateful for any help that you provide. Many providers find this challenge both fascinating and rewarding, but it may also be time consuming. Many of us who first started seeing PWCs found that we had to limit the number of such patients seen each week until we developed proficiency. It was also helpful to see patients regularly—perhaps every 2–4 weeks—and limit them to 1 or 2 problems at each visit. Many practitioners will reserve the last appointment of the day for such patients so that they can spend more time, if they wish.

A few practitioners will recognize that in order to treat ME/CFS one needs not only to understand the disorder itself, but one has to also develop proficiency in the many overlap disorders/co-morbidities that frequently complement ME/CFS (Table 1). This requires considerable knowledge and special skill sets that will challenge the practitioner and provide a lifetime of learning opportunities! Those who are drawn to care for ME/CFS full time frequently need to spend more time with patients, and therefore have to work outside the typical medical office schedule. Many find it necessary to privately contract with patients and charge an hourly fee-for-service rate, based on what the provider could have earned if he or she was seeing 4–6 patients per hour in a general practice setting. Since Medicare and Medicaid have no such provisions, it is necessary to opt out of such programs.

## Conclusion

ME/CFS is an “invisible illness” in that the patient appears normal despite tremendous hardship and impairments. For this reason these patients are frequently dismissed or mistaken as hypochondriacs. The patients greatly appreciate a provider who is knowledgeable about ME/CFS and who takes an interest in the disorder and is empathetic to their cause. Because ME/CFS is a chronic illness, regular follow-up and continuity of care is extremely important. Also, due to the many co-morbidities, one provider should assume the role of “gatekeeper” or “monitor” and provide a central source of information and prescriptions for the patient. This provider might follow-up with the patient on a monthly or quarterly basis; obtain periodic lab studies; insure that health maintenance is up-to-date; and maintain a repository of the patient's outside records and labs.

Many patients will seek information on the internet. Although much of the public information is misleading, there are several sites that can be recommended. These include:

1. Centers for Disease Control at http://cdc.gov/cfs
2. New Jersey Chronic Fatigue Syndrome Association at http://www.njcfsa.org
3. Massachusetts ME/CFS and FM Association at https://www.massmecfs.org
4. Treating CFS & FM: An Integrated Approach at http://www.treatcfsfm.org
5. A comprehensive Primer for physicians (2014 edition) can be found at: http://iacfsme.org/portals/0/pdf/Primer_Post_2014_conference.pdf.

## Author Contributions

The author confirms being the sole contributor of this work and has approved it for publication.

### Conflict of Interest Statement

The author declares that the research was conducted in the absence of any commercial or financial relationships that could be construed as a potential conflict of interest.
