# Supplementary material for: Initiating Care of a Patient With Myalgic Encephalomyelitis/Chronic Fatigue Syndrome (ME/CFS)
Source: Front Pediatr. 2019 Jan 23;6:415. doi: 10.3389/fped.2018.00415 (PMC6357921; doi:10.3389/fped.2018.00415)
Supplement: Supplementary file 1 [file Data_Sheet_1.docx]

**Appendix A: Canadian Consensus Criteria**

| **CLINICAL WORKING CASE DEFINITION OF ME/CFS** | |
| --- | --- |
| In order to be diagnosed with ME/CFS a patient must meet criteria for fatigue and post-exertional malaise; and/or fatigue, sleep disruption, and pain; *plus* have two or more neurological/cognitive manifestations; *plus* one or more symptoms from two of the categories of autonomic, neuroendocrine, and immune manifestations; *plus* adhere to Item 7 | |
|  | **1. Fatigue:** the patient must have a significant degree of new onset, unexplained, persistent, or recurrent physical or mental fatigue that substantially reduces activity level. |
|  | **2. Post-exertional malaise and/or fatigue:** there is an inappropriate loss of physical or mental stamina, rapid muscular and cognitive fatigability, post-exertional malaise and/or fatigue and/or pain and a tendency for other symptoms within the patient’s cluster of symptoms to worsen. There is a pathologically slow recovery period – usually 24 hours or more. |
|  | 3. **Sleep disruption:**  there is unrefreshed sleep or sleep quantity or rhythm disturbances such as reversed or chaotic diurnal sleep pattern |
|  | 4. **Pain:**  there is a significant degree of myalgia. Pain can be experienced in muscles and/or joint, and is often widespread and migratory in nature. Often there are significant headaches of new type, pattern, or severity. |
|  | 5. **Neurological / cognitive manifestations.**  Two or more of the following manifestations should be present: confusion, impairment of concentration and short term memory consolidation, disorientation, difficulty with information processing, categorizing and word retrieval, and perceptual and sensory disturbances, e.g. spatial instability and disorientation and inability to focus vision. Ataxia, muscle weakness, and fasciculations are common. There may be overload phenomena: cognitive, sensory -- e.g. photophobia, hypersensitivity to noise – and/or emotional overload, which may lead to “crash” periods or anxiety. |
|  | 6. **At least one symptom from two of the following categories:**  **Autonomic manifestations:** orthostatic intolerance -- Neurally Mediated Hypotension (NMH), Postural Orthostatic Tachycardia Syndrome (POTS), delayed postural hypotension; light-headedness; extreme pallor; nausea and irritable bowel syndrome; urinary frequency and bladder dysfunction; palpitations with or without cardiac arrhythmias; exertional dyspnea.  **Neuroendocrine Manifestations:** loss of thermostatic stability – subnormal body temperature and abnormal diurnal fluctuation, sweating episodes, recurrent feelings of feverishness and cold extremities; intolerance of extremes of heat and cold; marked weight change – anorexia or abnormal appetite; loss of adaptability and worsening of symptoms with stress.  **Immune Manifestations:** tender lymph nodes, recurrent sore throat, recurrent flu-like symptoms, general malaise, newer sensitivities to food, medications, and/or chemicals. |
|  | 7. **The illness persists for at least six months**: usually it has a distinct onset, although it may be gradual. Preliminary diagnosis may be considered earlier. Three months is appropriate for children. |
|  | **To be included**, the symptoms must have begun or have been significantly altered after the onset of this illness. It is unlikely that patient will suffer from all symptoms in criteria 5 & 6. The disturbances tend to form symptom clusters that may fluctuate and change over time. Children often have numerous prominent symptoms but their order of severity tends to vary from day to day. There are a small number of patients who have no pain or sleep dysfunction, but no other diagnosis fits but ME/CFS. A diagnosis of ME/CFS can be entertained when this group has an infectious disease type onset. Some patients have been unhealthy for other reasons prior to the onset of ME/CFS and lack detectable triggers at onset, or have more gradual or insidious onset. |
|  | **Exclude** active disease processes that explain most of the major symptoms of fatigue, sleep disturbance, pain, and cognitive dysfunction. |

Appendix B  **INTERNATIONAL / CDC CFS CASE DEFINITION***

NAME:______________________________________ DATE: ___________________

Step 1. **Exclude** alternative diagnoses and other causes of chronic fatigue.

Alternative diagnoses and other likely causes of chronic fatigue have been excluded by history, physical examination, exclusionary laboratory tests, and mental status examination.

There is no evidence of melancholic depression

Step 2. Does the patient have **chronic fatigue** that is both:

A perception of diminished and finite energy that with usual or normal activity demands a substantial change in work or school and the usual lifestyle?

New in onset (not lifelong) and has been persisting or relapsing for at least 6 months?

Step 3. Does the patient show **at least four classic symptoms**, which have been chronically or intermittently present for at least 6 months, make up a significant component of the illness, but have not predated the fatigue?

The new onset of cognitive dysfunction characterized by short term memory loss; word searching or poor recall; diminished comprehension or oral or written information; new difficulty in processing, maintaining, or expressing thoughts; possibly difficulty with sequencing of events or numbers, or difficulty with simple math (making change, keeping up finances).

Non-exudative pharyngitis. A non-exudative, “scratchy” or sore throat, frequent or relapsing in nature.

Lymphodynia or lymphatic soreness in at least two sites: anterior cervical, posterior cervical, axillary, or inguinal.

Muscle discomfort of a generalized nature. Flu-like myalgias or tenderness to touch (allodynia or “touch me not”).

Joint discomfort (arthralgias) usually migratory and involving large joints more than small joints.

Headaches of new onset or increased intensity, frequently retro-orbital or occipital and worsening with stress or exertion.

Sleep is disturbed and/or non-restorative.

It is helpful to subcategorize your patient.

Onset of illness

Abrupt onset over hours or days

Gradual or insidious onset

Severity of symptoms (clinician’s global impression)

Minimal – some symptoms especially with effort. Usually able to work.

Mild – mild symptoms and limitations, even at rest. May be able to work.

Moderate – moderate symptoms at rest, worse with effort. Unable to work.

Severe – often housebound or bedbound.

FINAL CLINICAL IMPRESSION

_____ Patient meets criteria for Chronic Fatigue Syndrome (Step 1 + Step 2 + > 4 symptoms)

_____ CFS is probable or possible but confounded by a concurrent medical or psychiatric

condition, or lacks sufficient criteria

_____ Idiopathic chronic fatigue. CFS unlikely or excluded by _______________________.

* Based on the international case definition criteria (Fukuda, et al. *Annals of IM*, 1994) and “A case definition for practitioners” (Lapp C, *Annals of IM*, 1995)

**Diagnostic Algorithm**

Based on International CFS Criteria (Fukuda, 1994, *Ann Int Med*)

Diagram 1

If ≤6 months: re-evaluate later; provide supportive treatment.

If lifelong: look for other causes, including depression.

No

Idiopathic Chronic Fatigue (ICF): Treat conservatively and follow periodically.

No

No

No

No

No other

explanation

YES

YES

YES

Diagnosis:

CHRONIC FATIGUE SYNDROME

Meets ≥4 of 8 Symptom Criteria

1. Impaired memory or concentration
2. Sore throat
3. Tender cervical or axillary lymph nodes
4. Muscle pain
5. Multi-joint pain
6. New headaches
7. Unrefreshing sleep
8. Post-exertional malaise

CFS is excluded if another plausible plausible explanation is found. Treat confounding conditions & re-evaluate as appropriate .

Idiopathic Chronic Fatigue (ICF): Treat conservatively and follow periodically.

History & Physical (including neurological & psychiatric evaluation); obtain exclusionary labs

Significantly affects lifestyle or ability to work

Chronic or relapsing fatigue ≥6 months but not lifelong
